# Glycoprotein is enough for sindbis virus-derived DNA vector to express heterogenous genes

**DOI:** 10.1186/1743-422X-8-344

**Published:** 2011-07-10

**Authors:** Wuyang Zhu, Jiangjiao Li, Li Tang, Huanqin Wang, Jia Li, Juanjuan Fu, Guodong Liang

**Affiliations:** 1 State Key Laboratory for Infectious Disease Prevention and Control, National Institute for Viral Disease Control and Prevention, Chinese Center for Viral Disease Control and Prevention, Beijing 100052, China

## Abstract

To investigate the necessity and potential application of structural genes for expressing heterogenous genes from Sindbis virus-derived vector, the DNA-based expression vector pVaXJ was constructed by placing the recombinant genome of sindbis-like virus XJ-160 under the control of the human cytomegalovirus (CMV) promoter of the plasmid pVAX1, in which viral structural genes were replaced by a polylinker cassette to allow for insertion of heterologous genes. The defect helper plasmids pVaE or pVaC were developed by cloning the gene of glycoprotein E3E26KE1 or capsid protein of XJ-160 virus into pVAX1, respectively. The report gene cassette pVaXJ-EGFP or pV-Gluc expressing enhanced green fluorescence protein (EGFP) or *Gaussia *luciferase (G.luc) were constructed by cloning *EGFP *or *G.luc *gene into pVaXJ. EGFP or G.luc was expressed in the BHK-21 cells co-transfected with report gene cassettes and pVaE at levels that were comparable to those produced by report gene cassettes, pVaC and pVaE and were much higher than the levels produced by report gene cassette and pVaC, suggesting that glycoprotein is enough for Sindbis virus-derived DNA vector to express heterogenous genes in host cells. The method of gene expression from Sindbis virus-based DNA vector only co-transfected with envelop E gene increase the conveniency and the utility of alphavirus-based vector systems in general.

## Findings

The concept that alphaviruses can be developed as expression vectors was first established by Xiong *et al*. [1]. Since then, Sindbis virus (SINV), a member of alphavirus, has been developed as vectors for the expression of heterologous gene [2-4], gene therapy and vaccine application [5-8]. Sindbis virus genome is a single strand of positive-sense RNA of approximately 12 kb which is capped at the 5' terminus and polyadenylated at the 3' terminus [9]. The 5' two-thirds of this RNA encode the nonstructural proteins (nsP1 through 4). The 3' one-third is initially translated as a polyprotein (NH2-C-E3-E2-6K-E1-COOH) that is processed co- and posttranslationally to produce the structural proteins (SPs) (capsid, El and E2). In infected cells, the virion structural proteins are translated from a subgenomic mRNA (26S RNA) and produced by transcription of genome-length complementary (minus) strand from a highly active subgenomic promoter. Since the nonstructural protein genes and the structural protein genes are expressed from two different mRNAs, they may be expressed independently of one another [10]. Thus, the high levels of expression of heterologous products are achieved when the viral structural genes are replaced by the heterologous coding sequences. Such recombinant vectors are self-replicating (replicons) and can be introduced into cells as naked RNA or plasmid DNA.

As a Sindbis-like virus, XJ-160 virus (GenBank No. AF103728) was isolated from a pooled sample of *Anopheles *mosquitoes collected in Xinjiang, China, in 1990 [11]. Recombinant plasmid pBR-XJ160 is an infectious full-length cDNA clone of XJ-160 virus, from which rescued virus BR-XJ160 can be obtained by transcription in vitro and transfection. The BR-XJ160 virus raised in BHK-21 cells was indistinguishable from the XJ-160 virus in its biological properties, including its plaque morphology, growth kinetics and suckling mouse neurovirulence [12]. On basis of pBR-XJ160, the effects of the substitutions of 169 Lys and 173Thr in nonstructural protein 1 (nsP1) as well as nsP2-726 Pro on the infectivity and pathogenesis of Sindbis virus have been investigated [13,14]. We have also confirmed the essential role of E2 glycoprotein, especially the domain of 145-150 aa, in Sindbis virus infection through the interaction with cellular heparan sulfate [15,16]. In addition, we have developed XJ-160 virus-based RNA vector system, including replicon vector pBRepXJ, a defective helper (DH) plasmid (pBR-H) and the packaging cell lines (PCLs) [17,18].

**Figure 1:**
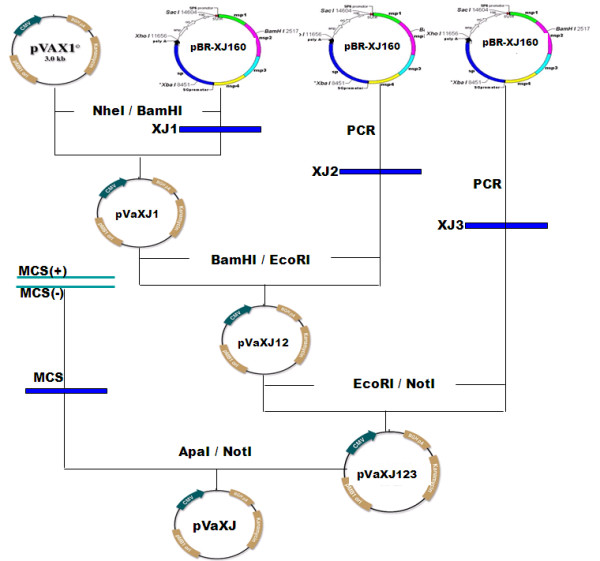
**Construction of pVaXJ vector derived from XJ-160 virus**.

The conventional approaches producing infectious Sindbis virus RNA and its derived complementary vectors were restricted primarily to in vitro transcription of cDNA clones from a bacteriophage RNA polymerase promoter, followed by transfection into permissive cells. Compared with this method, alphavirus replication can also be initiated by transfection of plasmid DNA [4,19]. In this case, full-length 5'-capped RNAs are transcribed in the nucleus using a polymerase II promoter and transported to the cytoplasm, the site of primary translation and RNA amplification. Therefore, there is no need for in vitro transcription and mRNA capping as required for the transfection of previously described Sindbis virus-derived RNA vectors. For the construction of XJ-160 virus-based DNA vector, the recombinant genome of XJ-160 virus was placed under the control of the human cytomegalovirus (CMV) promoter of the plasmid pVAX1 (Invitrogen, USA), in which viral structural genes were replaced by the sequence of multiple cloning site (MCS) to allow for insertion of heterologous genes (Figure 1). The virus non-structural gene sequence (1-7562nt), divided into three fragments XJ1 (1-2527nt), XJ2 (2527-5161nt) and XJ3 (5161-7562nt), was inserted into pVAX1 between unique *NheI *and *NotI *sites. The sequence of MCS under the control of subgenomic promoter contains several unique enzyme recognition sites, including *NotI, PvuI, FseI, PacI *and *AscI*. And the DNA vector derived from XJ-160 virus in this study was designated as pVaXJ. In addition, two DH plasmids were developed by cloning the gene of glycoprotein E3E26KE1 (8355-11297nt) or capsid protein (7563-8354nt) of XJ-160 virus into pVax1, respectively.

**Figure 2:**
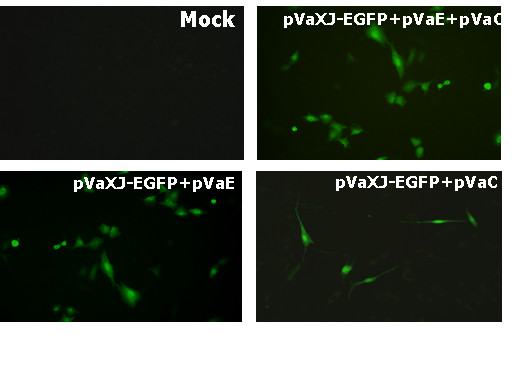
**Expression of EGFP on BHK-21 cells**. BHK-21 cells were cotransfected with pVaXJ-EGFP+pVaE+pVaC, pVaXJ-EGFP+pVaE, pVaXJ-EGFP+pVaC, respectively, and the expressions of EGFP were observed through scanned by an inverted fluorescence microscope (400×) at 24 hours postinfection. The BHK-21 cells without any transfection (Mock) were used as control.

To perform the qualitative and quantitative identification of pVaXJ, the report gene cassettes expressing either green fluorescence protein (EGFP) or *Gaussia *luciferase (G.luc) were constructed. In brief, the EGFP expression cassette pVaXJ-EGFP was constructed by inserting *EGFP *gene from the vector pEGFP-N1 (Invitrogen, USA) into the vector pVaXJ by digestion with *NotI *and *AscI*. Similarly, *G.luc *gene was amplified from pCMV-GLUC-1 vector (NEB, USA), and was inserted into pVaXJ with *FseI *and *AscI*.The G.luc expression cassette was designated as pV-Gluc. To investigate the functional activity of pVaXJ, and to determine whether both DH vectors are necessary to express report genes, BHK-21 cells were co-transfected with pVaXJ-EGFP+pVaE+pVaC, pVaXJ-EGFP+pVaE or pVaXJ-EGFP+pVaC, respectively. And the expressions of EGFP were observed through scanned by an inverted fluorescence microscope (400×). As can be seen in Figure 2, the green fluorescence was observed from all of three groups of BHK-21 cells at 24 hours post-cotransfection. In contrast, the control was negative. These results clearly demonstrated that XJ-160 virus-based DNA vector (pVaXJ) was able to efficiently express report genes by co-transfection with pVaE, pVaC or both of them, respectively. Importantly, the level of specific fluorescence from the BHK-21 cells co-transfected with pVaXJ-EGFP+pVaE was comparable to that produced by pVaXJ-EGFP+pVaC+pVaE, and obviously higher than that from the BHK-21 cells co-transfected with pVaXJ-EGFP and pVaC, suggesting that glycoprotein plays a more important role than capsid protein in expressing heterogenous gene from pVaXJ vector.

**Figure 3:**
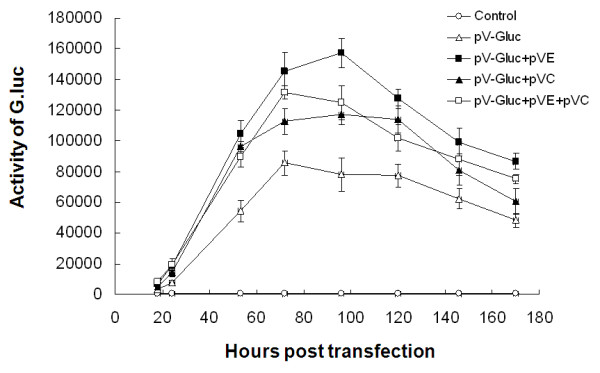
**Expression of G.luc on BHK-21cells**. BHK-21 cells were cotransfected with pV-Gluc, pV-Gluc +pVaE, pV-Gluc +pVaC or pV-Gluc +pVaE +pVaC, respectively. Gluc assay was performed by Gaussia Luciferase Assay System (Promega) at the indicated time postinfection.

For further comparing the necessity and potential application of structural genes, expression of *G.luc *gene was detected after BHK-21 cells were co-transfected with pV-Gluc, pV-Gluc+pVaE+pVaC, pV-Gluc+pVaE or pV-Gluc+pVaC, respectively. Gluc assay was performed by *Gaussia *Luciferase Assay System (Promega) following the manufacturer's instructions. As shown in Figure 3, *G.luc *gene was efficiently expressed in all groups of BHK-21 cells except that transfected with pV-Gluc plasmid, indicating that either glycoprotein or capsid protein is important for pVaXJ to express report genes. Interestingly, the activity of G.luc observed from the BHK-21 transfected with pV-Gluc+pVaE was much higher than that from the BHK-21 cells co-transfected with pV-Gluc+pVaE+pVaC, whose expression efficiency was similar to that of the BHK-21 cells transfected with pV-Gluc+pVaC. As mentioned earlier, the alphavirus particles are surrounded be a lipid bilayer containing envelop glycoprotein heterodimer. And the capsid subunits complex with the genome RNA to form a nucleocapsid acquiring the lipid envelop with embedded viral glycoproteins. In addition, the capsid protein of alphaviruses may be important factor in the inhibitionof host cell protein synthesis. Therefore, compared with capsid, glycoprotein plays a more important role in packaging virus particles or virus like particles, and is enough for Sindbis virus-derived DNA vector to express heterogenous genes in host cells.

DNA-based vector derived from alphavirus can be initiated by transfection of plasmid DNA [4,19]. In this strategy, full-length 5'-capped RNAs are transcribed in the nucleus using a polymerase II promoter, and transported to the cytoplasm. Then autocatalytic amplification of the vector, proceeds according to the Sindbis virus replication cycle and results in expression of the heterologous gene. Thus, as a double-stranded DNA, DNA-based vector can be directly transfected into the host cells dispensing with the process of in vitro transcription and mRNA capping, which is required for the transfection of previously described Sindbis virus-derived RNA vectors. In this study, we have constructed DNA-based vector pVaXJ from XJ-160 virus, which could efficiently express reporter genes in the transfected cells. The XJ-160 virus-derived DNA vectors described here increase the utility of alphavirus-based vector systems in general and also provide a vector with broad potential applications for genetic immunization.

The SINV-derived replicons lacking viral structural genes are suicide vectors, incapable of packaging into infectious particles and causing productive infection. To obtain viable particles, vector replicon can be packaged into infectious particles by cotransfection of cultured cells with a defective helper vector, which provides the virion structural proteins *in trans *[20]. Two principal strategies are being performed for construction of DH vector: (i) engineering recombinant plasmid that expresses viral subgenomic RNAs (NH2-C-E3-E2-6K-E1-COOH) and (ii) developing two DH vectors, one that expresses the capsid protein and a second that expresses the viral glycoproteins. High levels of expression of heterologous genes were achieved by using both of two methods. However, it is not clear which of the two SPs or both is necessary for SINV-derived vector to express heterologous coding sequences. In this investigation, based on the XJ-160 virus-derived DNA vector, our findings clearly prove that glycoprotein is enough for Sindbis virus-derived DNA vector to express heterogenous genes in host cells. The method of gene expression from Sindbis virus-based DNA vector only co-transfected with envelop E gene increases the conveniency and the utility of alphavirus-based vector systems in general.

## Competing interests

The authors declare that they have no competing interests.

## Authors' contributions

WZ carried out the molecular genetic studies, participated in the sequence alignment and drafted the manuscript. LT and JL carried out the immunoassays. HW, JL and JF participated in the sequence alignment and performed the statistical analysis. GL conceived of the study, and participated in its design and coordination. All authors read and approved the final manuscript.
